# Neural Associative Skill Memories for safer robotics and modelling human sensorimotor repertoires

**DOI:** 10.1162/NECO.a.1475

**Published:** 2025-12-22

**Authors:** Pranav Mahajan, Mufeng Tang, T. Ed Li, Ioannis Havoutis, Ben Seymour

**Affiliations:** 1 Nuffield Department of Clinical Neurosciences (NDCN) University of Oxford; 2 Department of Engineering Sciences University of Oxford; 3 Oxford Robotics Institute (ORI) University of Oxford; 4 Institute of Biomedical Engineering (IBME) University of Oxford; 5 Yale School of Medicine Yale University

## Abstract

Modern robots face a challenge shared by biological systems: how to learn and adaptively express multiple sensorimotor skills. A key aspect of this is developing an internal model of expected sensorimotor experiences to detect and react to unexpected events, guiding self-preserving behaviours. Associative Skill Memories (ASMs) address this by linking movement primitives to sensory feedback, but existing implementations rely on hard-coded libraries of individual skills. A key unresolved problem is how a single neural network can learn a repertoire of skills while enabling integrated fault detection and context-aware execution. Here we introduce Neural Associative Skill Memories (Neural ASMs), a framework that utilises self-supervised temporal predictive coding to integrate skill learning and expression using biologically plausible local learning rules. Unlike traditional ASMs, which require explicit skill selection, Neural ASMs implicitly recognise and express skills through contextual inference, enabling fault detection using ‘predictive surprise’ across the entire learned repertoire. Compared to recurrent neural networks trained via backpropagation through time, our model achieves comparable qualitative performance in skill memory expression while using local learning rules and predicts a biologically relevant speed-accuracy trade-off. By integrating fault detection, reactive control, and skill expression into a single energy-based architecture, Neural ASMs contribute to safer, self-preserving robotics and provide a computational lens to study biological sensorimotor learning.

## Introduction

Animals spend their lives learning, storing, and refining a repertoire of sensorimotor skills, which they must not only express adaptively but also use to detect abnormalities. To recognise an abnormal event, an animal requires an understanding of what normal actions ‘feel’ like. This corresponds to having a robust internal model of the body and its interactions with the world, i.e., a generative model that predicts the normal sensory consequences of actions. When reality deviates from these predictions, the resulting prediction error, or ‘predictive surprise’ (Clark, 2013; Friston, 2005), serves as a crucial signal. From a biological standpoint, this signal can indicate a sensorimotor conflict or an external perturbation (Shadmehr et al., 2010), or even bodily harm, such as an injury (Seymour and Mancini, 2020; Walters et al., 2023). The brain’s capacity to detect such violations of its internal priors is therefore not a secondary feature but a core component of safe and self-preserving behaviour.

Inspired by this principle, roboticists have sought to equip machines with analogous capabilities through Associative Skill Memories (ASMs) (Pastor et al., 2013, 2012). The core idea is to link motor commands, often represented by dynamic movement primitives (DMPs) (Ijspeert et al., 2013), with their expected sensory feedback. This allows a robot to detect ‘faults’, the engineering equivalent of injuries or perturbations, and react accordingly. However, traditional ASMs have a critical architectural limitation compared to their biological counterparts: they rely on a hard-coded, dictionary-like library of skills. Each skill is learned and stored as an independent module, requiring an external mechanism to explicitly select which one to execute. Consequently, fault detection is constrained to a single skill, failing to capture the integrated nature of biological motor repertoires.

This contrast between engineered libraries and integrated biological systems highlights a key unresolved question: how can a single, unified neural network learn a repertoire of sensorimotor skills in a biologically plausible manner? Such a system would need to solve two fundamental challenges that are handled seamlessly in the brain. First, it must be able to detect abnormalities with respect to all learned skills in the repertoire, without being explicitly told which skill is being performed. Second, it must be able to use sensory cues to contextually infer which skill memory is most appropriate to express in different scenarios.

This paper addresses this question by introducing Neural Associative Skill Memories (Neural ASMs), a framework that uses a temporal predictive coding network to learn an embodied generative model of a robot’s sensorimotor repertoire. We focus on how a sequential memory of sensorimotor observations can be learned from skill demonstrations, eschewing the separate problem of how optimal motor actions leading to these sequences are planned. Our central goal is to demonstrate how a single network, trained with self-supervised, local learning rules, builds upon the original concepts of Associative Skill Memories (Pastor et al., 2013, 2012). To this end, we provide basic demonstrations of three outcomes. First, the model performs fault detection across all learned skills in its repertoire. This is achieved by identifying abnormally high network energy, which is indicative of an out-of-distribution state with respect to the demonstration data. Second, it supports reactive correction by minimising proprioceptive prediction errors. Third, it facilitates the expression of different skills through contextual inference from early-stage cues, in a robotics simulation inspired by a human motor experiment (Sheahan et al., 2016). By doing so, we aim to provide a step towards safer, self-preserving robotics and offer a basic computational model for how the brain might learn, express, and monitor its own motor skills. Lastly, this study explores the foundational capabilities of a novel learning method involving local learning and inference within a temporal predictive coding framework. We demonstrate these capabilities using streamlined simulations, specifically fault detection in pick-and-place movements and perturbation compensation in point-to-point movements.

## Related Work

### Associative Skill Memories and Movement Primitives

**Figure 1:**
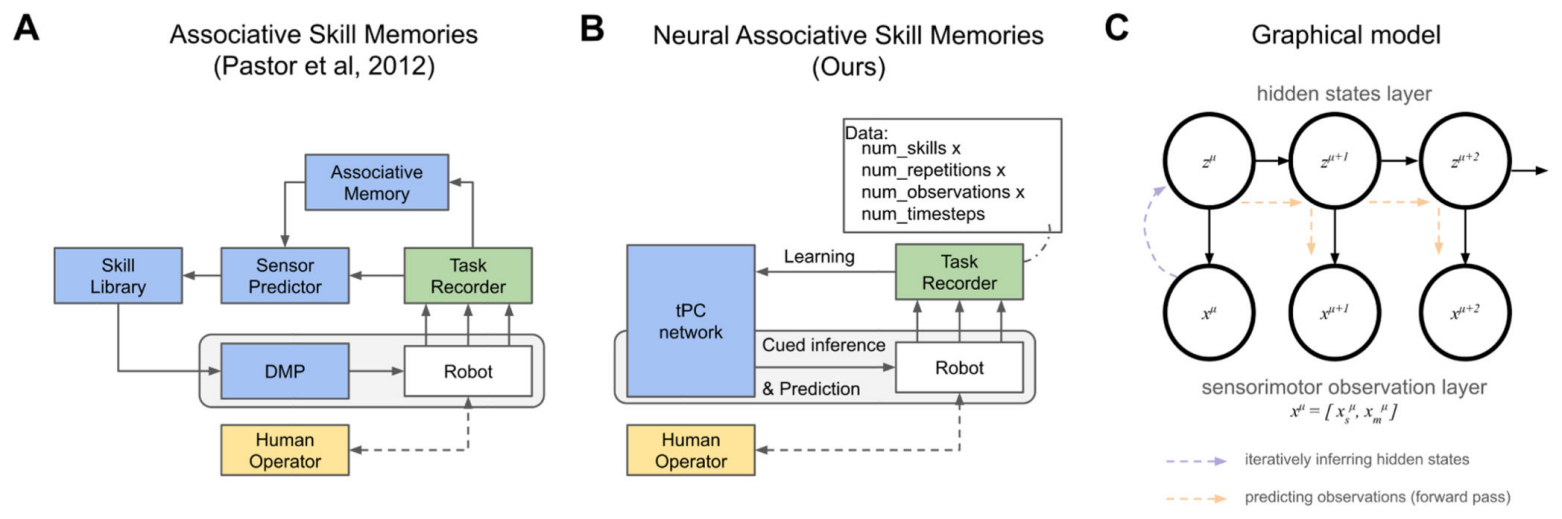
(A) Traditional ASMs (Pastor et al., 2012) use a modular, library-based architecture where each movement primitive and associated sensory statistics are stored separately. (B) Our Neural ASM approach replaces this with a single tPC network that learns multiple skill memories from demonstrations, where each demonstration is a time series of sensorimotor observations. (C) The underlying graphical model is a Hidden Markov Model (HMM), where hidden states (z) capture the dynamics and inferred context, and predict observations (x). Cued inference (purple arrow) sets the initial state from early observations, which then allows for offline prediction (orange arrows) of the sequence. While prediction can be a simple forward pass in an HMM, more complex generative models would require iterative energy minimisation.

The foundation of our work lies in Associative Skill Memories (ASMs) (Pastor et al., 2013, 2012), which extend Dynamic Movement Primitives (DMPs) (Ijspeert et al., 2013), a class of attractor-based models for generating stereotyped movements. The key idea of ASMs is to associate these motor primitives with their expected sensory feedback, enabling fault detection and reactive control. However, the standard ASM framework implements this using multiple handcrafted modules: an explicit skill library to store DMPs, a separate system to maintain sensory statistics for each movement, and a prediction module to find the closest match in the library (Fig. 1A). This modular, library-based architecture requires explicit skill selection and can only detect faults relative to one active skill at a time. Our work aims to overcome this limitation by learning a repertoire of skills within a single, unified neural network.

### Predictive Coding for Sensorimotor Learning

Our model is built on the principles of predictive coding (Clark, 2013; Friston, 2005; Rao and Ballard, 1999), an influential theory in neuroscience positing that the brain continuously generates predictions of sensory input and updates its internal model based on prediction errors. This self-supervised process has recently been proposed as a biologically plausible alternative to backpropagation (Song et al., 2024; Whittington and Bogacz, 2017, 2019) and has been applied to associative memory (Salvatori et al., 2021) and temporal sequence learning (Tang et al., 2024b). In robotics, predictive coding has been used for body state perception and multisensory integration from noisy information to filter internal state (Lanillos and Cheng, 2018). However, these models typically do not learn the dynamics of different movements, which is crucial for differentiating skills. Similarly, novelty detection (akin to abnormality detection in our work) has been demonstrated in static predictive coding networks by identifying inputs that generate high prediction errors (or energy) (Li et al., 2024), but not applied to temporal sequence learning or to the domain of neurorobotics. Our work extends these ideas by using a temporal predictive coding network to learn a dynamic model of a full sensorimotor repertoire, using the model’s energy as a natural signal for fault detection across all learned skills.

### Temporal Sequence Learning with Recurrent Architectures

Learning sensorimotor sequences has long been a domain of recurrent neural networks (RNNs). Influential work has used RNNs trained with backpropagation through time (BPTT) to generate complex sensorimotor sequences by learning associations between initial states and subsequent trajectories (Nishimoto et al., 2008; Nishimoto and Tani, 2004; Yamashita and Tani, 2008). A key insight from this research, particularly from Yamashita and Tani (2008), is the use of hierarchical structures with multiple timescales to capture complex temporal dependencies (MTRNNs). While powerful, these models typically rely on BPTT (Rumelhart et al., 1986), a non-local learning rule considered biologically implausible because it requires propagating error signals backwards through the network’s entire temporal history.

Our model, based on temporal predictive coding (tPC) (Millidge et al., 2024; Tang et al., 2024b), offers an alternative. Temporal predictive coding networks learn temporal dependencies using local, Hebbian-like updates that operate only between adjacent layers and time steps. This locality, while biologically plausible, is equivalent to BPTT truncated to one time step (Tang et al., 2024a). A benefit of tPC networks is that they integrate models of temporal sequence learning in the rich field of Bayesian inference. This allows us to use the notions of ‘energy’ of a model to formalise the concept of faults in this work. A key feature of our predictive coding framework is the iterative inference process used to converge on a hidden state at each time step. This process introduces an additional, faster timescale for online inference, distinct from the slower timescale of weight updates during learning. This mechanism shares some conceptual similarities with fast-weight RNNs (Ba et al., 2016) and error-regression in MTRNNs (Ahmadi and Tani, 2017). It further provides a candidate neural process for motor preparation, allowing contextual cues to shape a subsequent motor plan. These kinds of iterative inference from early-stage contextual cues further align with the hypothesis of the role of motor preparatory activity in setting the initial state of a dynamical system rather than explicitly representing movement parameters (Churchland et al., 2006a, 2010, 2006b; Cisek, 2006; Fetz, 1992).

### Computational Models of Homeostasis and Injury-related Behaviours

The concept of fault detection in our model aligns with computational theories of interoception and homeostasis, the processes by which the brain senses, predicts, and regulates the body’s internal state (Barrett, 2017; Seth, 2013). Our model adopts the view that the brain must infer its bodily state from multiple, often noisy, sensory signals (interoceptive, exteroceptive, and proprioceptive) to guide control (Seymour and Mancini, 2020). This contrasts with theories that assume direct access to (noise-free) internal states (Keramati and Gutkin, 2014) or rely purely on potentially noisy peripheral nociceptive signals to direct self-preserving behaviours (Walters et al., 2023). In this Bayesian inference-based view, the prediction errors arising from unexpected bodily signals drive an updated belief that the body is damaged, which in turn elicits protective behaviours (Mahajan et al., 2025; Seymour et al., 2023). Our model’s use of prediction errors to detect deviations from a learned ‘normal’ repertoire provides an analogous mechanism.

While other computational models have explored goal-directed homeostatic and interoceptive control (Mahajan et al., 2025; Tschantz et al., 2022), simulating interesting behaviours like investigating one’s injury to gain information about the injury state despite associated phasic pain (Mahajan et al., 2025), they typically rely on hand-crafted generative models or restrictive notions of homeostatic ‘setpoints’ (Keramati and Gutkin, 2014). This leaves two questions unanswered: (1) how can these ideas be extended to more complex robotics tasks, and (2) how can these computational-level theories be implemented with biologically plausible learning rules? Our work addresses both points by demonstrating that a generative model can be learned directly from demonstrations using local learning rules, providing a framework for fault detection and reactive control in a neuro-robotic system, while leaving goal-directed self-preserving behaviours as future work.

## Neural ASMs: A Theory Sketch

Neural Associative Skill Memories (Neural ASMs) replace the modular, library-based architecture of traditional ASMs with a single, unified network. This network is a temporal predictive coding (tPC) model (Millidge et al., 2024; Tang et al., 2024b) that learns a generative model of sensorimotor sequences from demonstrations (Fig. 1). The model has a hierarchical structure analogous to a Hidden Markov Model (HMM), where hidden states (*z*) predict observations (*x*) as well as their own future states. The sensory and motor observations are concatenated at the observation layer (*x*), similar to previous work (Nishimoto et al., 2008; Yamashita and Tani, 2008). The entire system learns by minimising a single energy function via local, Hebbian-like updates (see Methods, equations 1-4).

The model operates in two distinct phases: memorisation and recall. During memorisation, the network learns from demonstrated sensorimotor sequences. For each step in a sequence, it infers a hidden state and updates its weights, creating an attractor in its energy landscape that corresponds to that sequence (Salvatori et al., 2021; Tang et al., 2024b). This process allows the model to store multiple skill memories within a single set of weights.

During recall, the network retrieves a stored sequence from a partial cue. This begins with cued inference, where initial sensorimotor observations (e.g., the robot’s starting position and sensory state) are used to infer the corresponding hidden state (Fig. 1C, purple arrow). This inference process is analogous to motor preparation, where context sets the initial conditions for a movement (Churchland et al., 2010). Once the initial state is set, the network autonomously generates the predicted sensorimotor trajectory for the remainder of the skill (Fig. 1C, orange arrows). In this work, we use offline recall (Tang et al., 2024b), where the trajectory is generated ballistically after inferring the hidden state from a few early-stage contextual cues. Examples of such offline processes in biology include sequences guided from working memory (Mizes et al., 2023, 2024) or mental simulation, where trajectories are generated without producing actual movements (Jeannerod, 1994; Tani, 1996; Yamashita and Tani, 2008). An alternative, online recall, would involve continuous inference using incoming sensory data at each step, allowing for dynamic switching between learned skills, but this is not explored here.

Finally, the predicted sequence of motor commands (e.g., joint angles) from the Neural ASM serves as a high-level dynamical policy. This policy provides a reference trajectory to a low-level controller, which executes the movement and can make minor reactive adjustments online (cf. Schaal et al. (2007); Appendix 1, Fig. 7).

## Results

### Memorised skills are useful in fault detection and simple reactive correction

**Figure 2:**
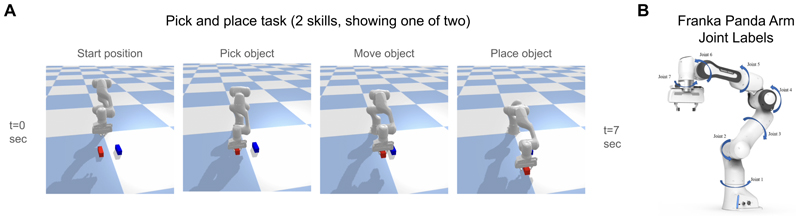
(A) Demonstration of Neural ASMs learning two pick and place skills in simulation. The sensorimotor sequences in the dataset used for learning from demonstrations are generated using predefined end-effector goals and use inverse kinematics to get joint angles. This is intended to be a proxy for teleoperation in simulation. (B) A schematic of Franka Panda arm joint labels (Adapted from Rogel et al. (2022), under CC BY 4.0 license. A title line was added.)

Having introduced Neural ASMs as a viable alternative to ASMs, we now demonstrate their core functionalities: fault detection and reactive fault correction in a simple simulation. We train a model to memorise two pick-and-place skills from demonstrations (Fig. 2A) and then test its ability to detect and react to simulated faults. The model uses implicit skill recognition during cued inference to retrieve the correct skill from initial observations using offline recall, unlike traditional ASMs, which require explicit selection of a movement primitive (Pastor et al., 2013, 2012).

**Figure 3:**
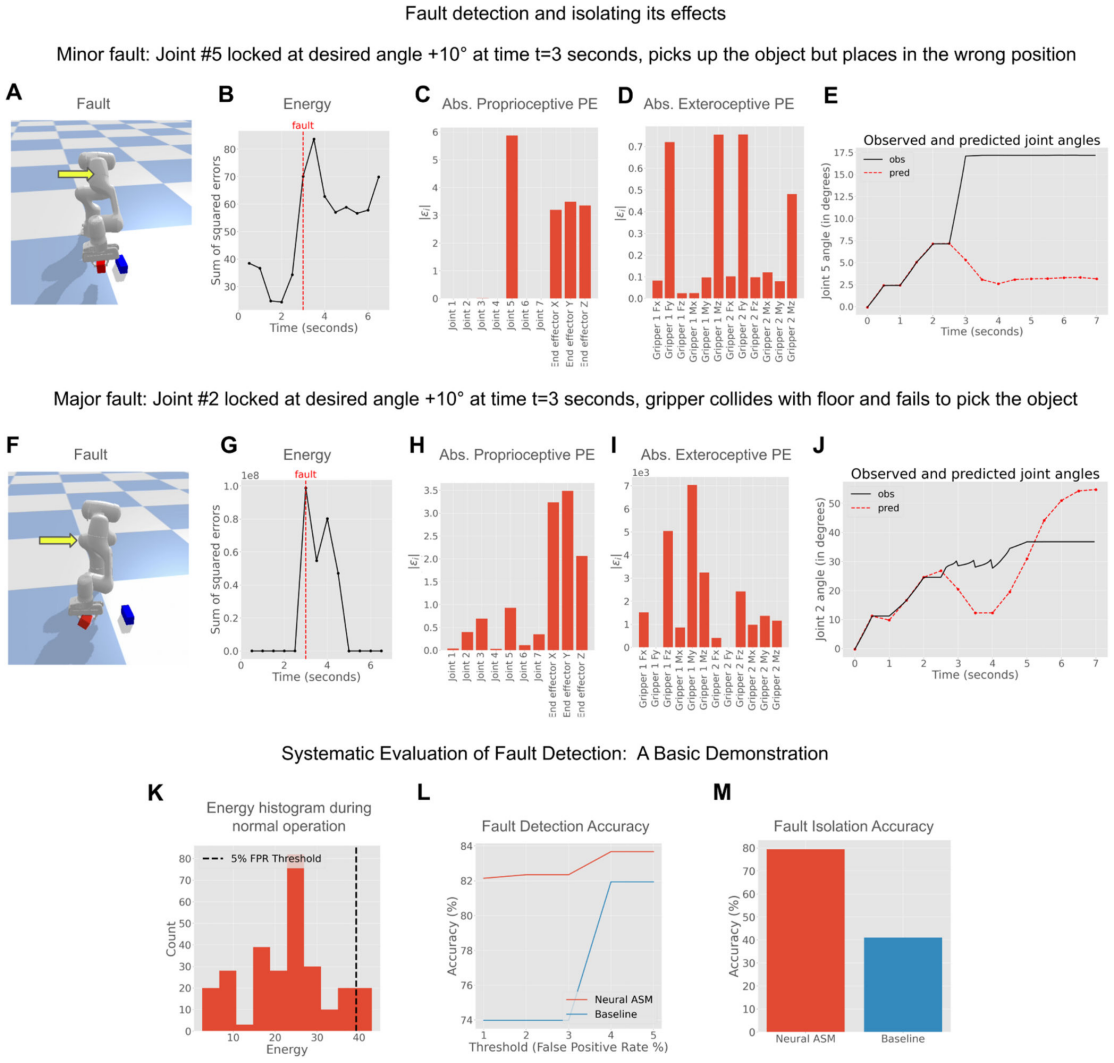
(A-E) Minor fault example with fault detection using energies and correct fault isolation using absolute prediction errors (denoted by abs. PE in figures) along with the joint angle time series. (F-J). Major fault example with fault detection using energies and incorrect fault isolation using absolute prediction errors (denoted by abs. PE in figures) along with the joint angle time series. The yellow arrows in Figure panels A and F point to the joints where the respective minor and major faults took place. The X-axis in panels C and H labels the proprioceptive sensors for joint angles (1-7, see Fig. 2B) and end-effector coordinates (X, Y, Z). The X-axis in panels D and I labels the exteroceptive sensors measuring X, Y and Z components of forces and torques at the two grippers. The Y-axis in panels C, D, H, and I represents the absolute prediction error (abs. PE). (K-M) A basic demonstration of the systematic evaluation of fault detection and isolation.

We first demonstrate fault detection qualitatively by measuring the network’s energy (i.e., sum of squared prediction errors). Fig. 3 shows two examples of a joint-locking fault. In a minor fault where joint 5 gets stuck (Fig. 3A), the network’s energy increases abnormally, successfully detecting the fault (Fig. 3B) using out-of-distribution detection on the network’s energy. The individual prediction errors also correctly identify joint 5 as the primary location of the fault (Fig. 3C-E). In a more severe fault where joint 2 gets stuck and causes a collision (Fig. 3F), the model again detects the fault via a spike in energy (Fig. 3G). However, in this case, the downstream effects of the collision cause larger prediction errors in other joints and sensors, illustrating that while the effects of a fault can be isolated, identifying the root cause remains a challenge (Fig. 3H-J). Please refer to the Methods section for more details on how the faults are simulated. A subtlety about Neural ASMs is that since the skill recognition is entirely implicit, the fault detection only depends on the current predictions, which in turn depend on the inferred context. This is unlike ASMs, which require knowing the explicit movement being performed to compare the observations to the corresponding signal statistics.

These qualitative examples of fault detection in different sensors are similar to Pastor et al. (2013, 2012). In addition, we perform a systematic evaluation of fault detection and isolation. For simplicity, we use percentile-based thresholding of the energy distribution during normal operation without faults to set the threshold for (out-of-distribution) fault detection. Fig. 3K shows the energy distribution during our pick-and-place task over 10 trials of normal operation for each skill, along with the 95th percentile threshold as an example, which is equivalent to a 5% false positive rate (FPR). We compare the performance of our Neural ASM against a baseline analogous to traditional ASMs that uses normalised Z-scores for error detection (see Methods for full details). By setting the detection threshold to correspond to a false positive rate (FPR) of 1–5%, we find that the Neural ASM correctly detects 82–83% of simulated faults, a modest improvement over the baseline’s 74–82% detection rate. However, the Neural ASM demonstrates a substantial advantage in fault isolation, correctly identifying the specific joint responsible for the fault in 79.5% of cases, whereas the baseline is only able to do so in 41% of cases. This concludes our basic demonstration of systematic evaluation, which can be extended to real-world robots with more realistic faults and comprehensive comparisons with alternate benchmarks in future work.

**Figure 4:**
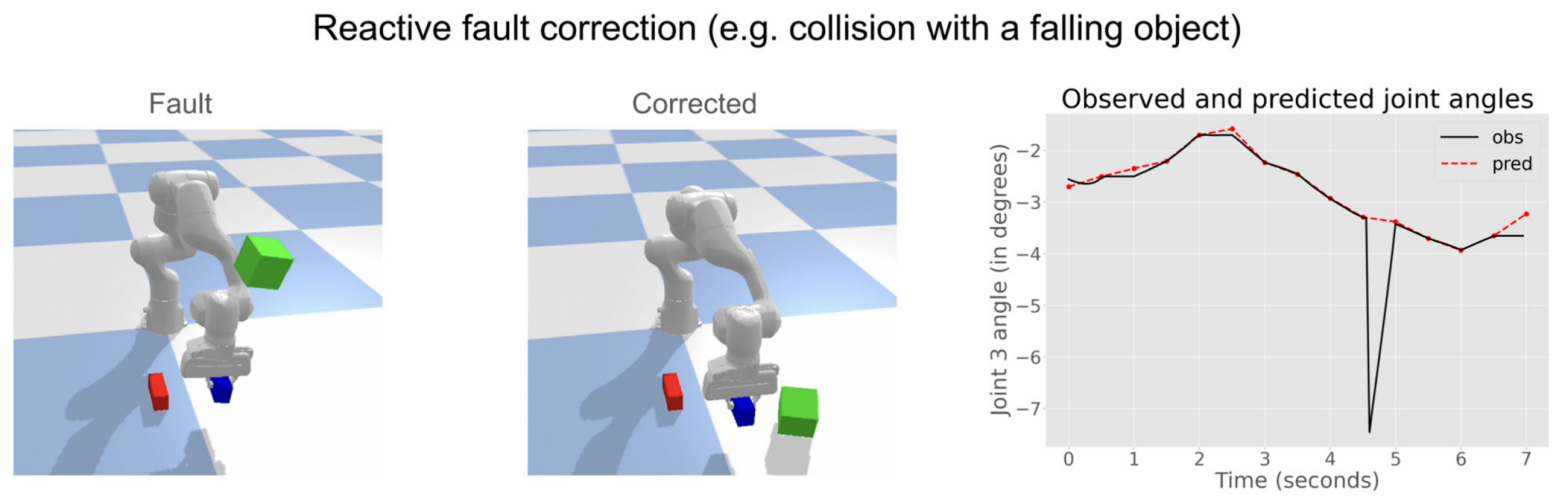
Demonstration of a fault resulting from a collision with a falling object. pThe fault is corrected reactively on the fly by having the low-level controller minimise proprioceptive prediction errors in joint configuration space using the proprioceptive predictions from Neural ASMs.

Lastly, in cases where faults can be corrected on-the-fly, Neural ASMs enable reactive correction. In ASMs, this is facilitated by DMPs, which themselves provide the reactive movement dynamics in end-effector space. In Neural ASMs, reactive correction is modelled by minimising proprioceptive prediction errors in either end-effector or configuration (joint) space, which is used for control. We demonstrate reactive fault correction by simulating a fault caused by a falling cube colliding with the robot, leading to temporary prediction errors. By minimising proprioceptive prediction errors in configuration-space based on predicted trajectory, the low-level controller automatically corrected for this disturbance (Fig. 4). In this simulation experiment, almost all faults can be corrected on-the-fly unless the grip strength is too weak and the object slips out of the grip due to the collision. A more systematic evaluation will require extending Neural ASMs to real-world robots along with alternative human-like methods for fault-correction, e.g. Collins et al. (2005), please see the Discussion section for more details.

In summary, this simple setup showcases that the core aspects of ASMs can be implemented using Neural ASMs: (1) fault detection, enabling the robot to halt and seek assistance for unresolvable faults, and (2) reactive fault correction, supporting real-time adjustments for robust, fault-tolerant control.

### Contextual inference in skill memory separation and expression

Having demonstrated the utility of Neural ASMs for self-preserving robots, we now demonstrate the role of contextual inference in the separation and expression of skill memories in our model. We utilise a robotics setup loosely inspired by Sheahan et al. (2016), who showed that motor planning of a follow-through motion, but not simply its execution, separates sensorimotor memories. However, we radically simplify the setup and eschew the optimal control that goes into arriving at the optimal trajectories, which compensate for the perturbations applied in their experiment. We rather assume that the appropriate sensorimotor sequences comprising the motor plan for each context are available in the demonstration dataset (for learning from demonstrations) and focus solely on under which conditions our model can or cannot learn and express these memories. We aim to explain and qualitatively simulate certain aspects of human motor behaviour in this robotics task. In doing so, we will also compare Neural ASM with less biologically plausible counterparts (baselines): sequence-to-sequence recurrent neural networks (RNNs) trained using backpropagation through time (BPTT). This simulation highlights the challenges shared by humans and machines that utilise neural networks to learn multiple skills, and would not usually arise if the system stored each skill independently in a library-like manner.

**Figure 5:**
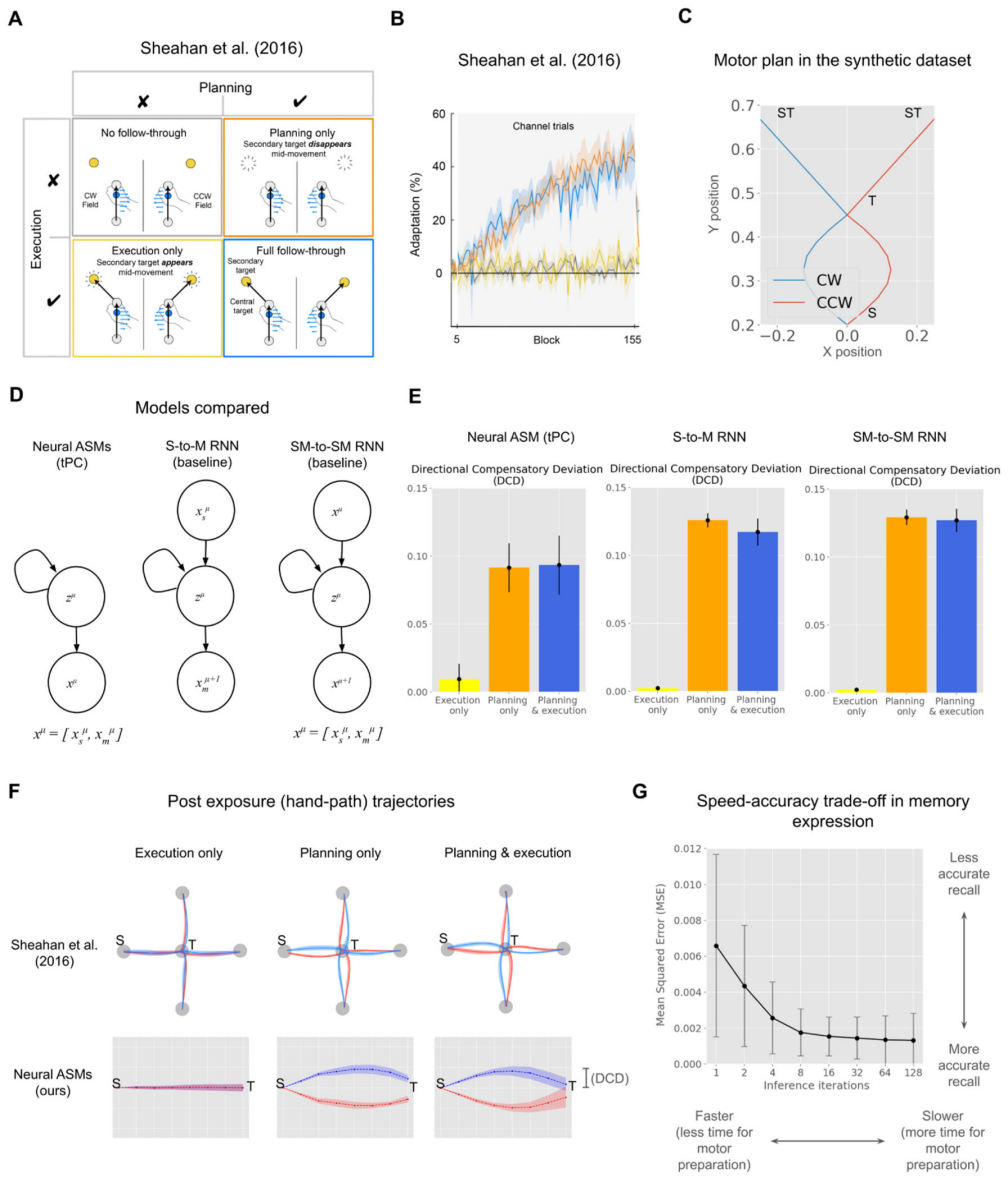
(A) Schematic from Sheahan et al. (2016) describing their experiment which inspires our robotics experiment. (Adapted under CC BY 4.0 license. Figure was cropped and a title line was added.) (B) Adaptation result from Sheahan et al. (2016)(C) Motor plan (end-effector positions) in the synthetic dataset, S: starting point, T: central target, ST: secondary target. Further details in Appendix 2, Fig. 8 (D) Simplified representations of our model and baseline RNNs. S-to-M RNN predicts the motor observations at the next discrete time step (µ + 1) using the sensory observations at discrete time step (µ) as input. SM-to-SM predicts the sensorimotor observations at the next discrete time step (µ + 1) using sensorimotor observations at the current time step (µ). (E) Results qualitatively replicated by our model, on par with baseline models. (F) Qualitative comparison of post-exposure (hand-path) trajectories (G) Speed accuracy trade-off, predicted in-memory expression. Here, demonstrated in offline skill recall.

Our simulation is inspired by the work of Sheahan et al. (2016), who investigated how motor planning affects the separation of opposing motor skills. In their study, participants reached towards a target while counteracting one of two opposing force fields. A visual cue, available either before or during the movement, indicated which force field was active. The crucial finding was that participants only learned to separate the two motor memories, producing distinct compensatory trajectories for each field, when the contextual cue was available before the movement, allowing for motor preparation (Fig. 5A-B). Therefore, in ‘Planning only’ and ‘Planning and Execution’ (full follow-through) conditions, participants could separate the memories, whereas they could not do so in the execution only condition, where the cue appeared mid-movement. This highlights the critical role of contextual inference during motor preparation for separating and selecting skill memories, a principle we test with our model.

To evaluate the capabilities of our framework, we compare the Neural ASM against two standard baseline models: a sensory-to-motor (S-to-M) RNN and a sensorimotor-to-sensorimotor (SM-to-SM) RNN (Fig. 5D). The crucial difference between these models lies in how they process contextual information. While our Neural ASM uses an iterative inference phase to model motor preparation from early cues, the baseline RNNs are trained with backpropagation through time (BPTT) and lack this distinct inference mechanism at runtime. All models are then tested on their ability to perform offline recall, generating a full trajectory from initial cues, with the specific architectures and training procedures detailed in the Methods section.

We find that in the ‘Planning only’ and ‘Planning and Execution’ conditions, all models successfully separate the two skill memories, but fail to do so in the ‘Execution only’ condition, where the contextual cue is unavailable during preparation. To quantify this, we measure the Directional Compensatory Deviation (DCD), which captures the (post-exposure) maximum trajectory deviation specifically in the direction that would correctly compensate for the expected perturbation (see Methods for formal definition). As shown in Figure 5E, the DCD is significantly greater than zero for the planning conditions across all models, indicating successful memory separation. In contrast, the DCD is near zero for the execution-only condition, confirming the models’ failure to express the correct motor plan. This result is further illustrated by the recalled trajectories in Figure 5F, which show distinct, opposing paths for the two contexts, qualitatively replicating the post-exposure hand paths reported by Sheahan et al. (2016). Sheahan et al. (2016) visualise their post-exposure hand path trajectories with 4 different starting points on the outside and ending in the central target, whereas we simplify when plotting our recalled motor plan in Fig. 5F. The same results can also be observed with alternative metrics such as mean square error in predicted trajectories in comparison to the motor plan (please see Appendix 3).

Interestingly, our model predicts a speed-accuracy trade-off in such ballistic actions, which is not predicted by baseline RNNs (Fig. 5G). Unlike baseline RNNs, our model has a cued iterative inference phase akin to setting the initial condition of the hidden state from multi-sensory integration. Since we hypothesise such iterative inference of the hidden state as a potential mechanism of motor preparation, fewer inference iterations mean a shorter or constrained time of motor preparation. We find that very few iterations may be inadequate for complete hidden state inference, leading to less accurate skill recall, which improves with an increase in inference iterations. This additional timescale in our model predicts such speed-accuracy recall during skill recall or expression. A similar speed-accuracy trade-off has been observed in the expression of habitual actions in human experiments by Hardwick et al. (2019), please refer to the Discussion section for more details.

We have demonstrated that our model can capture essential aspects of the results by (Sheahan et al., 2016) on the role of contextual inference in sensorimotor memory separation and expression in our simplified robotics setup, and is qualitatively on par with RNN baselines using non-local learning rules. Further, it predicts a speed-accuracy trade-off during skill memory expression, which is not predicted by baseline RNNs.

## Discussion

In this work, we introduced Neural Associative Skill Memories, a framework that leverages temporal predictive coding to learn an integrated generative model of a sensorimotor repertoire. Our results demonstrate that this approach, using biologically plausible local learning rules, can unify fault detection, reactive control, and contextual skill expression within a single network.

The model’s behaviour can be understood as a form of procedural memory, capturing the automatic, stimulus-driven recall of a well-learned sequence of actions (Dezfouli and Balleine, 2012; Éltető et al., 2022; Mizes et al., 2023; Simor et al., 2019). Our main finding is that the model’s energy, representing the sum of squared prediction errors, serves as a natural and effective signal for fault detection. This aligns with the neuroscientific view of the brain as a prediction machine, where ‘predictive surprise’ is fundamental for adaptive behaviour. While our fault-detection performance showed only a modest improvement over a simple baseline, we argue that the key contribution is conceptual. Unlike traditional approaches that require a separate module to compare observations against the sensory statistics of an explicitly selected skill, in our framework, this capacity emerges inherently from the model’s primary objective of predicting its sensorimotor stream.

Furthermore, we showed that the iterative inference process, where the model settles on a hidden state based on early cues, acts as a model of motor preparation and selects the appropriate skill memory to express. This provides a potential mechanistic account of motor preparatory activity, hypothesised to initialise a dynamical system without explicitly encoding movement parameters (Churchland et al., 2006a, 2010, 2006b; Cisek, 2006; Fetz, 1992). Iterative inference before plasticity refers to performing multiple inference steps to settle on a stable hidden state representation of the current observation before any synaptic weight updates are made. Such iterative inference before plasticity has also recently been demonstrated to have benefits over back-propagation in some biologically plausible tasks (Song et al., 2024). This provides a computational foundation for testing whether motor preparation (in terms of iterative inference) provides benefits in skill learning.

Further, a speed-accuracy trade-off arises from the model’s iterative inference mechanism, where fewer iterations (less preparation time) can lead to a less accurate initial state for recall. We note, that this trade-off does not arise in off-the-shelf recurrent neural networks. It directly links the time available for preparation (i.e., the number of inference iterations) to the accuracy of the recalled skill, a phenomenon observed in human habitual actions (Hardwick et al., 2019). When it comes to motor control, on one hand, Neural ASMs appeals to the concepts such as the equilibrium point hypothesis (Feldman and Levin, 1995) and passive motion paradigm (Mohan et al., 2019), aligning with the free energy principle (Friston, 2010; Friston et al., 2010). Whilst, on the other hand, Neural ASMs are also compatible with views by Schaal et al. (2007), where the dynamical systems policy sits atop the optimal control system, thus providing a possible unification of both approaches to motor control (Appendix 1, Fig. 7).

### Limitations

Our study serves as a proof of concept, and several limitations should be acknowledged. The experiments used a simplified robotics setup with a limited skill repertoire and basic evaluation metrics; future work should test the scalability, generalisation capabilities and robustness of Neural ASMs in more complex, dynamic environments. To simplify the demonstration, we simulate a limited repertoire of two pick-and-place skills, although the framework supports more. We also find that the rate of learning depends on the number of skills, consistent with observations in Howard et al. (2015) (see Appendix 4, Fig. 10). The reliance on learning from demonstrations means the model is not learning optimal control strategies itself, a key distinction from reinforcement learning approaches.

Furthermore, our experiment, inspired by Sheahan et al. (2016) is not an exact replica. We omit their "no follow-through" condition based on findings that no adaptation occurs from static cues alone (Gandolfo et al., 1996; Howard et al., 2012, 2013, 2015; Sheahan et al., 2016). The inability to learn to counteract perturbations from static cues is a limitation of the learning-from-demonstration paradigm itself, where optimal trajectories are provided, rather than our specific model. Nonetheless, this does not affect our central finding regarding how contextual inference supports the separation and expression of already learned skills.

On a framework level, further limitations exist. First, the model, under offline recall, relies on early-stage cues for contextual inference and cannot adapt to goal changes mid-trajectory. Online recall could be used for re-recognition or switching between alternative skills in the repertoire. This would involve inferring the hidden states using the sensorimotor observations at each time step, allowing the model to continuously infer the skill it is performing and then predict the sensorimotor observations for the next time step. However, it would not be able to perform novel goal-directed planning to change goals or to achieve certain preferred sensory observations. Second, our model concatenates sensorimotor channels into a single input vector, whereas a more neurally plausible architecture might involve separate, interacting pathways for different modalities. Contextual selection of the correct skill would require motor and sensory observations to be connected to a shared hidden state. Third, the capacity of the tPC network is constrained by its local-in-time learning rule, which restricts its ability to learn complex, long-horizon sequences. Finally, all Associative Skill Memory models (Pastor et al., 2013, 2012) assume that skill-related movements are stereotyped; high sensory variability would make the learned predictions unreliable.

### Future Work

The limitations discussed above highlight several promising directions for future research. A critical next step is to test the scalability and robustness of the Neural ASM framework in more complex, dynamic environments with a larger repertoire of skills. For robotic applications, improving reactive controllers, for instance by incorporating principles from passive dynamic machines (Collins et al., 2005), would be a valuable step towards more energy-efficient and human-like reactive behaviours.

At a systemic level, incorporating goal-directed planning would require disentangling sensory and motor modalities. The concatenated input structure, similar to (Nishimoto et al., 2008; Yamashita and Tani, 2008), would need to be replaced by a more neurally plausible architecture with separate, interacting pathways for different modalities. Future work can aim to integrate ideas on goal-directed planning to achieve a preferred sensory state similar to (Matsumoto et al., 2022). One could potentially use a sensory tPC network for inferring the internal states from sensory observations and a "model inversion" of a motor tPC network to implement goal-directed motor planning, exploiting the duality of Bayesian inference and optimal control (Doya, 2021). This would allow for more flexible behaviour, such as adapting to mid-trajectory goal changes, and is an avenue for future work. Additionally, enabling Neural ASMs to track posterior variance, e.g., via Monte Carlo Predictive Coding (Oliviers et al., 2024) would make them viable as planning-capable world models. To overcome the limited temporal credit assignment of the current tPC network, one could investigate multi-timescale tPC architectures (Yamashita and Tani, 2008) or alternative biologically plausible approximations to BPTT using memory traces, such as e-prop (Bellec et al., 2020).

### Conclusion

In conclusion, this work presents a biologically inspired framework for sensorimotor learning in robots which unifies skill learning, fault detection, and contextual expression within a single predictive coding network. Neural ASMs further offer a plausible approximation of how associative sensorimotor memories might be implemented in the brain using local learning rules. The conceptual contribution of this work provides a step towards creating more adaptive, self-preserving robots and offers a tractable computational testbed for exploring theories of biological motor control.

## Methods

### Model algorithm

The sequential memory in Neural ASMs is modelled using a temporal Predictive Coding (tPC) network (Millidge et al., 2024; Tang et al., 2024b). Predictive coding models learn in a self-supervised fashion with the aim of best predicting the incoming input based on its own learned generative model. The model evaluates the actual input against its prediction by determining the difference in activity in the respective error neurons and minimises these ‘errors’ through adjusting neural activities and synaptic weights, which corresponds to the processes of inference and learning, respectively (Bogacz, 2017; Clark, 2015).

**Figure 6:**
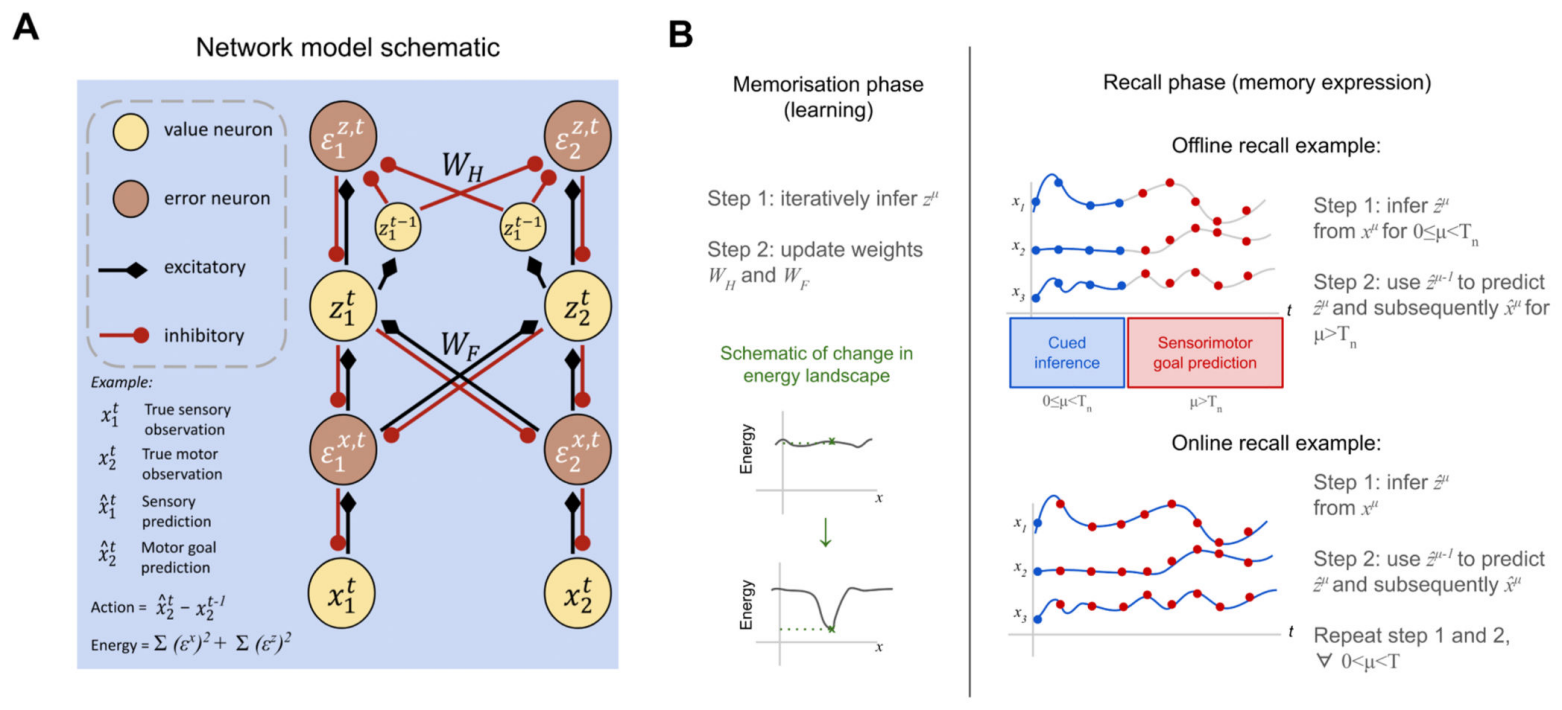
(A) Neural network implementations of temporal predictive coding (tPC) model used in Results for simulations. The network is illustrated with a single sensory and motor observation input for didactic purposes (though in reality, the network will have multiple sensorimotor observations as inputs). (B) The model learns in the memorisation phase in a self-supervised manner, and the weight updates change the energy landscape to store these memories as attractors in the energy landscape (which is crucial in recognising memorised skills). During the recall phase, the learned memories are expressed and can be either done offline (e.g. ballistic actions) or in an online manner where ground-truth observations are utilised to provide online feedback through model inversion at each step.

In mathematical terms, the task of neural ASMs can be seen as learning a sequence of sensorimotor observations (such as motor coordinates and associated sensory events like haptic feedback, etc) ${\left(x^{t}\right)}_{t=0}^{T}.$ This can be reduced to learning the dynamics in these sensorimotor observations for each skill, i.e. learning to associate each **x**^*µ*^ with the next **x**^*µ*+1^ (*µ* = 0, 1, …, *T* − 1). We use a 2-layer tPC model, whose underlying graphical structure is that of a Hidden Markov Model (HMM). The lower layer of the tPC predicts the sensorimotor observations (**x**^*µ*^) and the upper layer predicts the next hidden state (**z**^*µ*+1^). This predictive processing account loosely models the hierarchical processing of raw sensory inputs by the neocortex, where hidden value neurons **z**^*µ*^ models the brain’s internal neural responses to the sequential sensory inputs **x**^*µ*^ (Fig. 6A).

The working of the tPC model in neural ASMs can be divided into two stages: (1) memorisation and (2) recall (Fig. 6B). During memorisation, tPC tries to minimise the sum of squared errors at step *µ*, with respect to the weights and the hidden activities:

(1) $$F_{\mu }\left(z^{\mu },W_{H},W_{F}\right)=||z^{\mu }-W_{H}f\left({\hat{z}}^{\mu -1}\right)|{|}_{2}^{2}+x^{\mu }-W_{F}f\left(z^{\mu }\right)|{|}_{2}^{2}$$

where **W**_*H*_ governs the temporal prediction in the hidden state, **W**_*F*_ are the weights governing predictions from **z**^*µ*^ to **x**^*µ*^, with ${\hat{z}}^{\mu -1}$ being the hidden state inferred at the previous time-step.

During memorisation, the model first infers the hidden representation of the current sensorimotor observational input **x**^*µ*^ by:

(2) $${\dot{z}}^{\mu }\propto -\frac{\partial F_{\mu }\left(z^{\mu },W_{H},W_{F}\right)}{\partial z^{\mu }}=-\varepsilon^{z,\mu }+f^{\prime }\left(z^{\mu }\right)⊙W_{F}^{⊤}\varepsilon^{x,\mu }$$

where ⊙ denotes the element-wise product between two vectors, and *ε*^**z**,*µ*^ and *ε*^**x**,*µ*^ are defined as the hidden temporal prediction error $z^{\mu }-W_{H}f\left({\hat{z}}^{\mu -1}\right)$ and the top-down error **x**^*µ*^ − **W**_*F*_ *f* (**z**^*µ*^), respectively. After **z**^*µ*^ converges, **W**_*H*_ and **W**_*F*_ are updated following gradient descent on *F*_*µ*_:

(3) $$\text{Δ}W_{H}\propto -\frac{\partial F_{\mu }\left(z^{\mu },W_{H},W_{F}\right)}{\partial W_{H}}=\varepsilon^{z,\mu }f{\left({\hat{z}}^{\mu -1}\right)}^{⊤}$$

(4) $$\text{Δ}W_{F}\propto -\frac{\partial F_{\mu }\left(z^{\mu },W_{H},W_{F}\right)}{\partial W_{F}}=\varepsilon^{x,\mu }f{\left(z^{\mu }\right)}^{⊤}$$

which are performed once for every presentation of the full sequence. Importantly, the converged **z**^*µ*^ is then used as ${\hat{z}}^{\mu }$ for the memorisation at time-step *µ* + 1.

After memorisation (learning) is completed, the model enters the recall stage, where all weights no longer change and the previously learned memories are expressed in response to certain cued input observations (also referred to as queries *q*). Note that during the recall phase, the observation layer has no access to the correct patterns for the complete movement. Instead, it needs to dynamically change its value to retrieve the memories to predict these sensorimotor observations. The sequential memories are recalled or expressed using the learned weights **W**_*H*_ and **W**_*F*_. The loss thus becomes:

(5) $$F_{\mu }\left(z^{\mu },{\hat{x}}^{\mu }\right)=||z^{\mu }-W_{H}f\left({\hat{z}}^{\mu -1}\right)|{|}_{2}^{2}+||{\hat{x}}^{\mu }-W_{F}f\left(z^{\mu }\right)|{|}_{2}^{2}$$

where ${\hat{x}}^{\mu }$ denotes the activities of value neurons in the observation layer during recall. Both the hidden and observation layer value neurons are updated to minimise the loss. The hidden neurons will follow similar dynamics specified in Eq. 2, whereas the observation layer neurons are updated according to:

(6) $${\dot{\hat{x}}}^{\mu }\propto -\frac{\partial F_{\mu }\left(z^{\mu },{\hat{x}}^{\mu }\right)}{\partial {\hat{x}}^{\mu }}=-\varepsilon^{x,\mu }$$

and the converged ${\hat{x}}^{\mu }$is the final retrieval.

In the case of sequential memory, there are two types of recall, offline and online. In case of offline recall, first ${\hat{z}}^{\mu }$ is iteratively inferred from *T*_*n*_ cued input ground-truth observations or queries *q* = **x**^*µ*^, where *µ* = (0, 1, …, *T*_*n*_) (here, 0 ≤ *T*_*n*_ *< T*). Once ${\hat{z}}^{\mu }$ is converged, it is used to recall ${\hat{x}}^{\mu }$ for *µ > T*_*n*_. In case of online recall, we query the model with *q* = **x***µ* (ground-truth), use the query to infer ${\hat{z}}^{\mu },$ and then use ${\hat{z}}^{\mu }$ for the recall the next time step *µ* + 1 for *µ* = 0, 1, …, *T* − 1. This distinction is important in our results; here, we only present offline recall results for skill memory expression in ballistic movements.

### Network training details

The network details for the tPC network are as follows: The number of hidden units was 256, and the number of sensorimotor observation units depended on the task. The learning rate for the weight updates was 10^−4^ and the default learning iterations were 1000 per skill, trained with a batch size of 1. The iterative inference learning rate for hidden state update was 10^−2^ with default inference iterations set to 100. The same hyperparameters were used for all simulation experiments. Kaiming uniform initialisation was used in hidden layers for all networks.

The S-to-M RNN and SM-to-SM RNN are sequence-to-sequence RNNs. They used the same number of hidden units, learning rate, learning iterations and batch size at the tPC network. They did not have iterative inference functionality, like the tPC network. The input unit size is the number of sensory observations, and the output unit size is the number of motor observations. The skill memory expression experiment trains the RNNs to predict the output sequence using inputs only from the first time step to mimic the offline recall used in tPC.

The sensorimotor sequences were Z-score normalised for each observation channel before being provided as inputs to all neural networks. Outputs were again unnormalised to original units before movement.

### Experimental setup and robot simulation details

#### Task 1: Fault Detection and Reactive Correction

In our simulations, the Neural ASM operates at 2 Hz, guiding an underlying low-level position controller operating at 40 Hz. The demonstration dataset was generated in simulation by providing end-effector goals, from which joint angles were calculated using inverse kinematics and sensory observations were recorded. This process acts as a proxy for teleoperation. The experiment used 10 repetitions for each of two distinct pick-and-place skills. Each repetition is a sensorimotor sequence of 15 time steps (at 2 Hz), involving 25 observations (including desired joint angles, end-effector positions, gripper states, and sensed forces and torques), with realistic noise from the simulation process.

The minor fault is simulated as follows: the joint 5 overshoots the desired goal by 10 while attempting to pick up the object (*t* = 3 seconds) and gets stuck in that configuration for the remaining duration (Fig. 3A). The major fault is simulated as follows: the joint 2 overshoots the desired goal by 10 and gets stuck while attempting to pick up the object, which results in the arm colliding with the floor (Fig. 3F).

For the systematic evaluation of fault detection, we use percentile-based thresholding of the model’s energy distribution during normal operation to establish a detection threshold, as illustrated in Fig. 3K. This method allows us to control for the false positive rate (FPR); for instance, a 95th percentile threshold corresponds to a 5% FPR. We then measure accuracy on 980 simulated fault trials, created by systematically varying the locked joint (1 to 7), the time of fault (1 to 6 seconds in 0.5s steps), and the degree of joint angle overshoot (-15 to +15 in 5 steps). Fault isolation accuracy is measured as the proportion of trials where the joint with the highest absolute prediction error matches the simulated fault location.

We compare our model’s performance against a simple baseline analogous to traditional ASMs (Pastor et al., 2012), which relies on stored signal statistics. This baseline computes normalised errors for each observation channel using Z-score normalisation $\left(\varepsilon =\left(x_{i}^{t}-{\bar{x}}_{i}\right)/\sigma_{i}\right).$. The sum of squared normalised errors is then used for fault detection, and the channel with the maximum absolute normalised error is used for fault isolation. We acknowledge that this represents one possible implementation of a statistics-based approach and that other methods may exist, and a comprehensive comparison with alternate methods is left for future work.

#### Task 2: Contextual Skill Expression

Inspired by the experimental paradigm of Sheahan et al. (2016), we designed a simulation to test the model’s ability to separate skill memories based on contextual cues. It is important to note that our goal was not to model the learning of optimal trajectories to counteract perturbations, but rather to assess if the model could learn and express distinct, pre-defined motor plans from demonstrations under different contextual conditions. The synthetic dataset, therefore, provides these optimal compensatory trajectories directly. For each skill, the data includes end-effector positions, joint angles calculated via inverse kinematics, and a one-hot coded visual cue representing the context (see Appendix 2 for plots). The sensory and motor sequences used in the skill memory expression are presented in Appendix 2. We did not model the "no follow-through" condition from the original study, for reasons explained in the Discussion.

We compared our Neural ASM against two baseline recurrent neural network (RNN) models (Fig. 5D): a sensory-to-motor (S-to-M) RNN and a sensorimotor-to-sensorimotor (SM-to-SM) RNN, a discrete-time variant of the model used in Nishimoto et al. (2008). Unlike our tPC-based model, these baselines are trained using backpropagation through time (BPTT) and lack an iterative inference phase. To test skill expression from preparation, all models were evaluated using an offline recall procedure, generating the full trajectory based only on the inputs from the first time step. To parallel the human experiment, we trained all networks on each condition using 6 different random seeds for 1200 learning trials, and post-training recall was averaged over 24 trajectories.

To quantify memory separation, we introduce the Directional Compensatory Deviation (DCD) metric, which, unlike a simple absolute deviation, accounts for the direction of movement. DCD is defined as the maximum deviation of the recalled end-effector trajectory perpendicular to the straight line connecting the start (S) and target (T) points, projected onto the axis of correct compensation for a given context (e.g., leftward for a clockwise field). A positive DCD value indicates that the trajectory deviates in the appropriate direction to counteract the expected force field, while a value near zero indicates a failure to express the correct motor memory. This metric serves as a direct proxy for the directional adaptation measured by Sheahan et al. (2016).

## Supplementary Material

Supplementary Materials

## Data Availability

The code is available at https://github.com/PranavMahajan25/NeuralASMs
