# Supplementary material for: Neural Associative Skill Memories for safer robotics and modelling human sensorimotor repertoires
Source: Neural Comput. Author manuscript; Available in PMC 2026 Jan 29. (PMC12746714; doi:10.1162/NECO.a.1475)
Supplement: Supplementary Materials [file EMS212191-supplement-Supplementary_Materials.pdf]

Appendix 1: Unifying view on dynamical systems vs optimal control

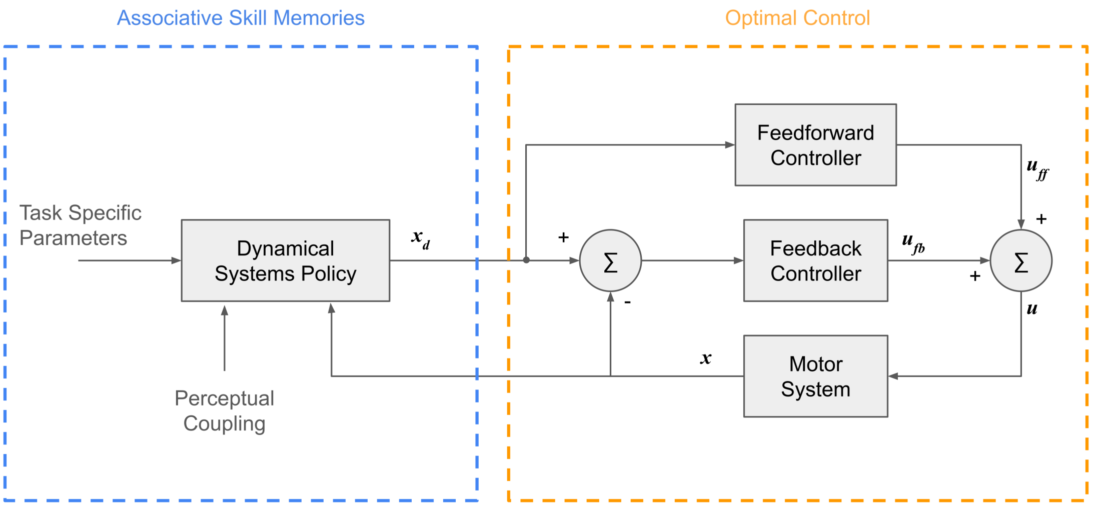

Figure 7

Unifying view proposed by (Schaal et al., 2007), where the dynamic systems policy from Associative Skill Memories can employ an optimal control-based low-level controller.

Appendix 2: Demonstration data used in skill memory expression task

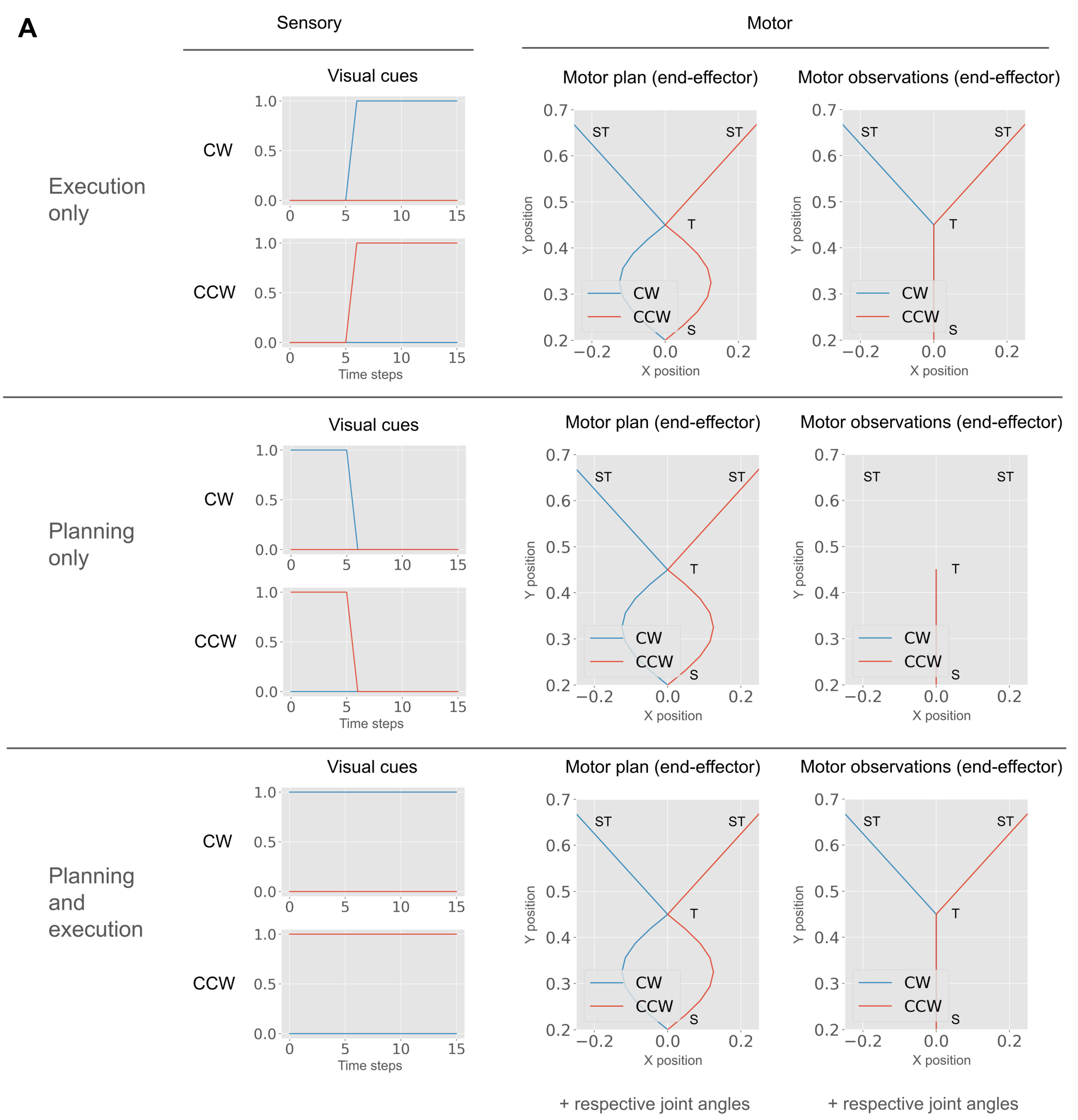

Figure 8

Plots of the sensory and (hypothetically optimal) motor sequences used for demonstrations in robot experiments on skill memory expression inspired by Sheahan et al. (2016).

Appendix 3: Additional results in the skill memory expression task

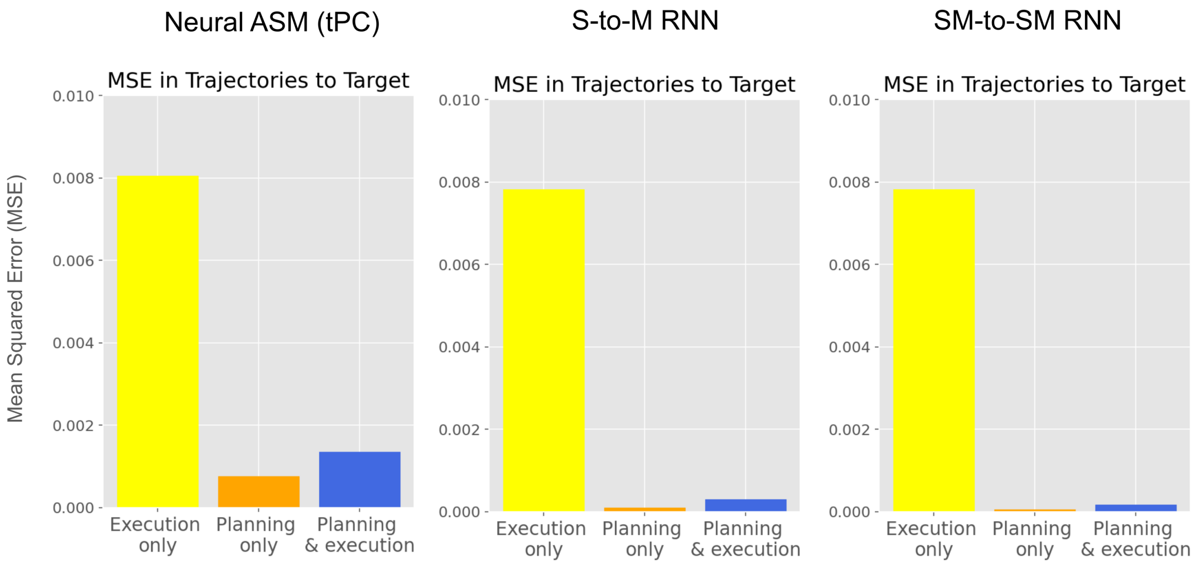

Figure 9

Mean Squared Errors (MSE) in trajectories to the target as an alternate metric for quantifying the results in the skill memory expression task, inspired by Sheahan et al. (2016). MSE will show an inverse relationship to the DCD metric that we constructed or seen in the trajectory plots of Sheahan et al. (2016), i.e. failure to separate the skill memories results in low DCD and high MSE and vice versa.

Appendix 4: Learning rate varies with number of skills

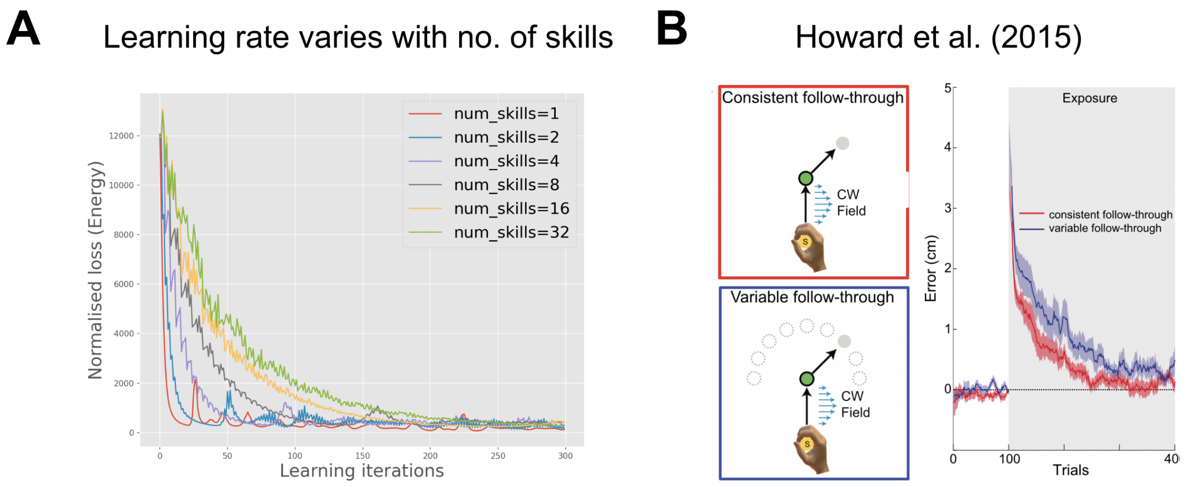

Figure 10

Lesser the number of distinct skills to memorise, the faster is the learning rate of our model, as seen in energy (normalised loss) over epochs. This potentially explains why consistent follow-throughs improve learning rates as variable follow-throughs can split the learning into different skill memories rather than a single memory, as observed by Howard et al. (2015). (Adapted from Howard et al. (2015), under CC BY 4.0 license. Figure was cropped and a title line was added.)
